# Isolation, identification and phenotypic and molecular characterization of pathogenic *Vibrio vulnificus* isolated from *Litopenaeus vannamei*

**DOI:** 10.1371/journal.pone.0186135

**Published:** 2017-10-18

**Authors:** Tao Teng, Liguo Liang, Kai Chen, Bingwen Xi, Jun Xie, Pao Xu

**Affiliations:** 1 Wuxi Fisheries College, Nanjing Agricultural University, Wuxi, Jiangsu, China; 2 Key Laboratory of Freshwater Fisheries and Germplasm Resources Utilization, Ministry of Agriculture, Freshwater Fisheries Research Center, Chinese Academy of Fishery Sciences, Wuxi, Jiangsu, China; Chang Gung University, TAIWAN

## Abstract

The morphology and the drug sensitivity of the strain GYX2014-1 isolated from the hepatic pancreatic tissue of moribund *Litopenaeus vannamei* were evaluated by conventional culture characteristics, physical and chemical characteristics, and molecular biology methods. Detection of extracellulase and hemolysin activity shows that the isolated GYX2014-1 has protease, lipase, gelatinase activity, but none of amylase, or lecithinase activity. The 16S rRNA gene (GenBank accession number: KT781675) was analyzed, and a phylogenetic tree analysis showed that the isolated pathogen was most closely related to *V*. *vulnificus* (GenBank accession number: NR 118570)—a match of more than 99%. The phenotypic traits and molecular biology of isolated bacteria, determined their identity as *Vibrio vulnificus* (*V*. *vulnificus*). In addition, artificially infected *L*. *vannamei* with *Vibrio vulnificus* appeared with the same disease symptoms as those of naturally infected shrimp. Drug sensitivity tests showed that *V*. *vulnificus* is highly sensitive to fosfomycin, cefradine and sinomin, and was resistant to penicillin, amikacin and kanamycin. This experiment is the first to separate *V*. *vulnificus* from *L*. *vannamei*, and the findings of this study can be used as a reference for disease control and health management.

## Introduction

*Penaeus vannamei Boone*, scientific name *Litopenaeus vannamei* [1], commonly known as *white-leg shrimp* or *white shrimp*, are eurythermal euryhalinous tropical shrimp. Their appearance resembles *Chinese shrimp*, *Penaeus merguiensis*. Their body length measures up to 24 cm and they have a thin, light gray in color shell with no body markings. *L*. *vannamei* are consumed for their highly nutritious and delicious meat encased in a thin shell, as a result they are a lucrative crustacean species to farm worldwide [2–4]. Since 1994–1995, *L*. *vannamei* have been successfully artificially bred in China. Because of their rarity in naturally occurring environments they are widely popular among Asian shrimp producers—accounting for more than 77% of the farmed shrimp production in China [5]. However, frequent disease such as *Vibriosis*, are responsible for the mortality of shrimp culture to the great distress of farmers [6–8]. For example, the immune system of *L*. *vannamei* was destroyed by *Vibrio Parahaemolyticus*, causing illness and death [9].

*Vibrio vulnificus*, is a prominent invasive species within the *Vibrio spp*. [10], also known as *Marine Vibrio*, they are Gram-negative, halophilic [11] and facultatively anaerobic bacteria [12]. They are zoonotic pathogens [13], commonly found in warm water, mainly growing in fish and shellfish [14]. As one of three majors pathogenic *Vibrios* (*V*. *cholera*, *V*. *vulnificus*, and *V*. *Parahaemolyticus*), *V*. *vulnificus* can cause human diseases such as gastroenteritis, septicaemia [15], acute lethal disease of *V*. *vulnificus* sepsis [16], and the animal disease *Nibea albiflora* [17].

The main artificial breeding model of *L*. *vannamei* is fish and shrimp polyculture cultivated in higher-place pond and soil pond, and the key to shrimp culturing lies in shrimp disease prevention and control. In May 2016 at a *L*. *vannamei* farm in the south of Jiangsu Province, the cultivated shrimp succumbed to mass illness. it was believed that the main infectious agent was from *Vibrio sp*., and suspected pathogenic bacteria *V*. *vulnificus* was isolated from diseased shrimp. At present, research surrounding *L*. *vannamei* mainly concern feed nutrition [18] and control of disease [19], but the specific study of *V*. *vulnificus* infection has not been reported. As *L*. *vannamei* farming increase in popularity research on the disease treatment is becoming more and more important. Therefore, in this experiment, *L*. *vannamei* carrying *Vibrio spp*. were thoroughly investigated. We studied the phenotypic characteristics of the isolated bacteria, their main biological characteristics, extracellular enzymes produced, their 16S rRNA gene sequence and conducted phylogenetic analysis which detected the sensitivity of the bacteria to antibacterial drugs in order to provide a reference point for further research on effective inspection of *L*. *vannamei* for *Vibrio* and basis for prevention and control of disease epidemiology.

## Materials and methods

### Isolation and purification of bacteria

10 newly infected *L*. *vannamei* were selected. The liver and pancreas were sectioned to pieces under sterile operation, and tissue fluid was inoculated on TSA, 5% sheep blood TSA and BHI agar plate medium, and incubated at 28°C for 24h. 10 colonies were randomly selected and repeatedly streak-plated. After the artificial infection, a pure fatal culture strain was obtained for GYX2014-1 and preserved at 4°C on TSB agar medium.

### Detection of extracellular enzyme activity

The isolates GYX2014-1 were tested for the production of extracellular products such as lipase, lecithin enzyme, protease and amylase. They were inoculated onto LB agar plate containing yolk liquid (10%), Tween 80 (1.0%), skim milk (10%), starch (1%), and observed in 30°C for 24h, after which a hydrolysis ring can be directly observed on the plate containing egg yolk, Tween 80 and skim milk.

They were considered extracellular enzyme positive if a transparent ring formed around the colony [20]. I_2_-KI solution (Gram staining with Lugol's solution) was added on tablet containing starch before observing, if the bacteria produced amylase, starch is decomposed, thus, there would be no reaction with iodine and the culture medium around the colony becomes transparent. If the bacteria did not produce amylase, the starch in the medium would react with iodine and a purple colour would be observed [21]. The detection of gelatinase activity was detected through a biochemical reaction tube via the gelatin liquefaction method, and cultured at 30°C for 24h after inoculation. Colonies were observed overnight after being placed at a temperature of 4°C in the refrigerator, if the gelatin liquified, colonies were considered gelatinase positive.

### Artificial infection experiment

The experiment was conducted in strict accordance with the US National Institutes of Health (NIH Publication No. 85–23, revised 1996) and the regulations for use of animals in experimentation. The study protocol was approved by the Research Ethics Committee, Wuxi Fisheries College of Nanjing Agriculture University (S1 Table).

Healthy *L*. *vannamei* were challenged with pure GYX2014-1 cultures to test bacterial pathogenicity. *L*. *vannamei* were raised for three days before being infected by bath immersion. The bacteria were inoculated aseptically in TSB liquid medium at concentrations of 1×10^8^, 5×10^7^, 1×10^7^, 5×10^6^ and 1×10^6^ CFU·mL^-1^ and incubated for 24 h at 160 rpm. Each concentration was divided into three groups of 20 shrimp each and sterile TSB liquid medium was established as the control group at the same time. After inoculation, *L*. *vannamei* were fed in an isolation tank. Dead shrimp were selected as standard isolates to study GYX2014-1 pathogenicity.

### Identification of bacteria

#### Physiological and biochemical characteristics

The bacterial isolates were systematically evaluated [22] for the metabolism of glucose (alcohol and glycosides), organic acid salt utilization, H_2_S generation, glucose gas, nitrate reduction and other tests, in order to determine their physiological and biochemical characteristics.

#### 16S rRNA gene sequence analysis and phylogenetic analysis

Pure isolated bacterial cultures were inoculated in nutrient broth, incubated at 30°C for 24h and collected at 12000 rpm for 1min. DNA was extracted and used as a PCR template using the UNIQ-10 column bacterial genome extraction kit (Sangon Biotech (Shanghai) Co., Ltd., China), according to the manufacturer’s instructions.

The specific test methods and parameters of 16s rRNA gene sequence analysis from isolates are mentioned in Teng’s method [23], the reaction products were electrophoresed and observed with a gel imaging system. Gene sequencing of the PCR products were performed by Sangon Biotech (Shanghai) Co., Ltd., China.

The NCBI BLAST database was used to analyse sequence homology for extracted 16S rRNA gene sequences. The software ClusterX2.0 and strain sequences obtained from the GenBank database of nucleic acid sequences which showed high similarity to extracted 16s rRNA were used to perform multiple sequence alignment, following which a phylogenetic tree was constructed using the adjacent method by MEGA5.1 software and tested by bootstrap (1000 repetition).

#### Drug sensitivity test

Sensitivity to commonly used antimicrobial drugs of *V*. *vulnificus* strains was determined by routine agar diffusion method (K-B) under aseptic technique. After determining the bacterial concentration to be (1×10^7^)-(1×10^8^) CFU·mL^-1^, the ring of inhibition was used to categorize the unknown bacteria as either susceptible or resistant [24]. Drug sensitive paper was purchased from Hangzhou Binhe Microbial Co. Ltd., China.

## Results

### Colony morphology

Colony morphology is an important characteristic of the bacteria identification. In this study, we have obtained an advantage growth of monoclonal colony after separation and purification, which is smooth, circular, transparent, central bulge, 2mm in diameter. Colonies were Gram-negative.

### Extracellular enzyme activity

The phenotypic characteristics of *Vibrio spp*. vary in living organisms, and their rapid and accurate identification has been a problem [25]. Strain GYX2014-1 has protease, lipase, gelatinase activity, but not amylase or lecithinase activity.

### Ecological characteristics of isolates

The results of the physiological and biochemical tests are shown in Table 1, and the data in the table are from *Bergey’s Manual of systematic bacteriology*, *Second edition* [26] and *Common Bacterial System Identification Manual* [22].

**Table 1:** Physiological and biochemical characteristics of the isolated strain GYX2014-1.

Characteristic	Strain		Characteristic	Strain	
	GYX2014-1	*V*. *vulnificus* *		GYX2014-1	*V*. *vulnificus* *
**Glucose**	+	+	**Nitrate (reduction)**	+	+
**Lactose**	+	d	**Nitrate (gas)**	-	•
**Maltose**	+	•	**Acetate**	+	•
**Mannitol**	+	d	**Tartaric acid salt**	+	•
**Mannose**	+	+	**Mucic acid**	+	•
**Sucrose**	-	-	**Indole**	+	•
**L- Arabia sugar**	-	-	**Trehalose**	+	+
**Arabitol**	-	•	**Raffinose**	-	•
**Xylose**	+	-	**Fructose**	+	•
**Xylitol**	-	•	**Melibiose**	-	-
**Galactose**	+	+	**Cellobiose**	+	+
**Melezitose**	-	•	**Peptone Water**	-	•
**Sorbitol**	-	-	**Glucose ammonium**	+	•
**Sorbose**	-	•	**Ornithine decarboxylase**	+	-
**Dulcitol**	-	•	**Lysine decarboxylase**	+	•
**Erythritol**	-	•	**Arginine decarboxylase**	-	•
**Amygdalin**	+	•	**Arginine hydrolase**	-	-
**Rhamnose**	-	-	**hydrogen sulfide**	-	•
**Dextrin**	+	•	**Gelatin**	+	+
**Inositol**	-	-	**Acetamide**	+	•
**Salicin**	+	•	**OF tube**	F	F
**Urea**	+	•	**Potassium cyanide**	+	•
**Bile esculin**	+	+	**α- methyl -D-glucoside**	-	•
**Beta galactosidase**	+	+	**Semisolid**	+	•
**Malonate**	+	+	**Glucose (acid)**	+	+
**Simmons citrate**	-	+	**Glucose (gas)**	-	-
**Adonitol**	-	•	**Motility**	+	+

Note:

^“+”:^ Denotes positivity;

^“-”:^ Denotes negativity

* not recorded

### The analysis results of 16S rDNA gene sequence

**Fig 1:**
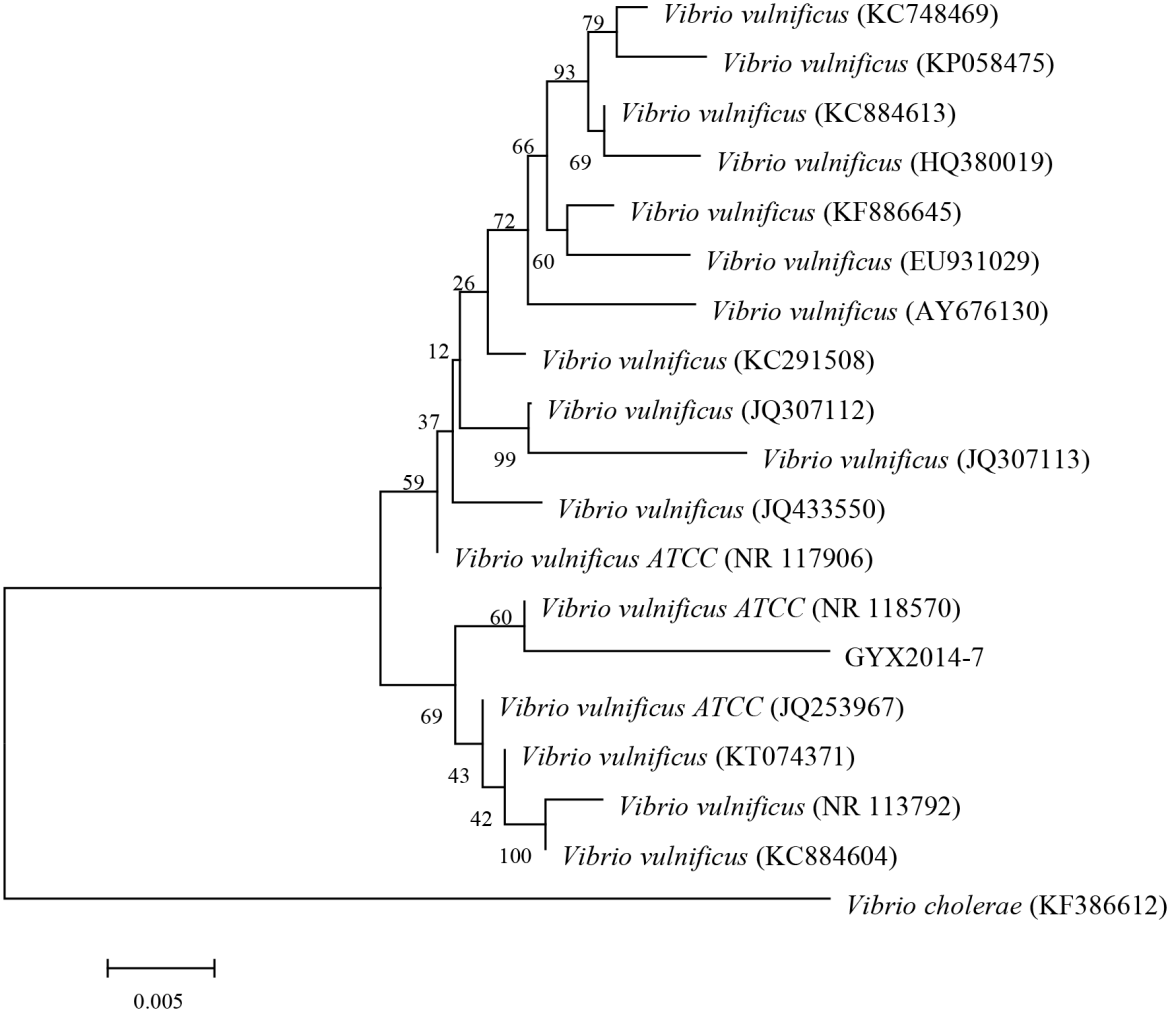
Phylogenetic tree of the strain GYX2014-1 based on 16S rRNA gene sequence.

The length of the PCR product was 1448 bp (GenBank accession number: KT781675). The results retrieved were 16s rRNA gene sequences of *Vibrio spp*., with 99% similarity to *V*. *vulnificus*, and 94% similarity to *V*. *cholerae* (GenBank accession number: KF386612). Constructed phylogenetic tree analysis showed that the isolated bacteria were most closely related to *V*. *vulnificus* (GenBank accession number: NR 118570) and the farthest from *V*. *cholerae* (GenBank accession number: KF386612) (Fig 1 and S1 Text).

### The drug sensitivity test

In accordance to NCCLS criteria [27], the diameters of the inhibition zones were measured from 43 different drug sensitivity tests using GYX2014-1. Antibiotic resistance was determined and the results are shown in Table 2. The results confirmed that the strains were sensitive to fosfomycin, cefradine, sinomin and 17 other antibiotics. They were moderately sensitive to rifampicin, tobramycin, ciprofloxacin and 7 other antibiotics but resistant to penicillin, tobramycin, amikacin, kanamycin and 19 other antibiotics.

**Table 2:** Drug sensitivity test results of the isolated strain GYX2014-1.

Drug name	Drug content (μg/ Piece)	Bacteriostatic ring (mm)	Sensitivity	Drug name	Drug content (μg/ Piece)	Bacteriostatic ring (mm)	Sensitivity
**Penicillin**	10	7	R	**Cephalothin**	30	7	R
**Ampicillin**	10	7	R	**Doxycycline**	30	19	I
**Carbenicillin**	100	11	R	**Norfloxacin**	10	7	R
**Oxacillin**	7	12	R	**Nalidixic acid**	30	25	S
**Cefuroxime**	30	23	S	**Acetyl spiramycin**	30	7	R
**Cefazolin**	30	7	R	**Maddie mycin**	30	11	R
**Cefoperazone**	75	25	S	**Cefotaxime**	30	28	S
**Ceftazidime**	30	26	S	**Enrofloxacin**	5	7	R
**Ceftriaxone**	30	27	S	**Ciprofloxacin**	5	19	I
**Rifampicin**	5	15	I	**Lomefloxacin**	10	7	R
**Streptomycin**	10	7	R	**Enoxacin**	10	21	S
**Kanamycin**	30	12	R	**Levofloxacin**	5	23	S
**Amikacin**	30	7	R	**Aztreonam**	30	31	S
**Gentamicin**	10	7	R	**Minocycline**	30	24	S
**Tobramycin**	10	16	I	**Clarithromycin**	15	42	S
**Novobiocin**	30	13	R	**Cefradine**	30	25	S
**Tetracycline**	30	13	R	**Cefamandole**	30	28	S
**Vancomycin**	30	7	R	**Ofloxacin**	5	7	R
**Erythromycin**	15	15	I	**Fosfomycin**	200	32	S
**Lin Ke mycin**	2	16	I	**Neomycin**	30	18	I
**Cotrimoxazole**	23.7/1.25	21	S	**Teicoplanin Lalin**	30	26	S
**Fleroxacin**	5	25	S				

Note: S: The diameter of inhibition zone including drug diameter 7mm; Denotes high sensitivity (d≥20mm); I: Denotes moderate sensitivity (15 mm≤d≤19 mm); R: Denotes low or no sensitivity (7 mm≤d≤14 mm).

### Artificial infection experiment

*L*. *vannamei* infected by artificially injecting different doses of isolated bacterium showed different mortality rates, while the control group had no death during the experimental observation period (Table 3 and S2 Table). The mortality rate was 100% in *L*. *vannamei* injected with 1×10^8^ CFU·mL^−1^, whereas only 50% of the *L*. *vannamei* injected with 1 × 10^7^ CFU·mL^−1^ died. All shrimp in the control group injected with 0.85% normal saline survived. Bacteria from artificially infected *L*. *vannamei* showed identical morphological characteristics and physiochemical properties to the original inoculum.

**Table 3:** Results of the artificial infection experiment using by the isolate strain GYX2014-1.

Group	GYX2014-1/CFU·mL^-1^	Deaths/tail	Trials/tail	Mortality/%
**The test group**	1×10^8^	20	20	100
	5×10^7^	16	20	80
	1×10^7^	10	20	50
	5×10^6^	0	20	0
	1×10^6^	0	20	0
**The control group**	Normal saline	0	20	0

## Discussion

Bacteria of *Vibrios spp*. are ubiquitous and the *Vibrio* genus contains numerous kinds of species relevant to food safety [28]—their presence which pose a big threat to public health. Many seafood associated disease outbreaks reported worldwide are caused by *Vibrio spp*. such as *V*. *alginolyticus* (3–19%) and *V*. *harveyi* (1–7%) which were identified in the coastal farms of India [29]. Foodborne pathogen *V*. *parahaemolyticus* was found in aquatic products from hypermarkets in Shanghai [30] and Beijing [31], and the most hazardous *Vibrio* species—*V*. *cholera* was detected in *L*. *vannamei* [32, 33]. Eating food contaminated with *Vibrios* increases the risk of infection, and even causes severe food poisoning to humans. Therefore, the study of *Vibrios* isolated from diseased aquatic animal is of great importance.

*V*. *vulnificus* is one of the most dangerous bacteria [34], humans are infected with *V*. *vulnificus* by eating raw seafood or having contact with seawater via the gastrointestinal mucosa by swallowing or broken skin contact. Based on differences in biochemical, genetic, and serological tests and preferred host for infection, *V*. *vulnificus* are divided into three kinds of biological type: biological type I produce indole, biological type II does not produce indole, biological type III [35] all are pathogenic humans. Among them, biological II type is an important pathogenic bacterium in shrimp, and especially infectious in eel. Therefore, *V*. *vulnificus* is an important pathogenic bacterium of "Human and shrimp disease" and have received wide attention in medical science and shrimp disease science. A deeper understanding of the microbiological characteristics of *V*. *vulnificus* can assist in prevention and clinical treatment of bacterial diseases infected by *V*. *vulnificus*.

The dominant bacteria isolated from artificially infected, diseased *L*. *vannamei* was confirmed as pathogens, which are highly pathogenic to *L*. *vannamei*. The onset of symptoms was the same as the naturally infected shrimp confirming as the identity of the bacteria which caused the devastating deaths of *L*. *vannamei* in the Gaoyou area of Jiangsu Province. In this paper, the drug sensitivity test results were generally consistent with previous research [11], which showed *V*. *vulnificus* to be sensitive to cefotaxime, ceftazidime, and cotrimoxazole. However, tetracycline is confirmed as the most effective antibiotic for use in systemic septicemia infections [36]—inconsistent to the results of this paper. This may be due to strains obtained from different sources and different experimental animals used. In actual shrimp production, antibiotic combinations are used together to overcome disease.

Upon considering the histopathological features of dead *L*. *vannamei*, the morphology, enzymatic activity, physiological and biochemical characteristics 16S rRNA sequence and sensitivity to antibiotics by bacterial isolates, the identity of bacteria extracted from *L*. *vannamei* was judged to be highly pathogenic *V*. *vulnificus*. This is the first time *V*. *vulnificus* from moribund *L*. *vannamei* was extracted while the separation and identification of *V*. *vulnificus* has been performed in other aquatic animals such as eel [37], stone flounder [38] and *Synechogobius hasta* [39]. The establishment of a rapid detection method to identify and judge *V*. *vulnificus* disease timely and effectively is required. Other researchers have found that polysaccharides added in the feed could improve the immune function of *L*. *vannamei* [40], which provides the premise and the foundation for assessment of the probability of an outbreak, and provides a reliable basis for further disease prevention and control measures in cultured aquaculture species.

## Supporting information

S1 Table Animal Care and Use Certification of Wuxi Fisheries College, Nanjing Agricultural University. (PDF)

S2 Table Artificial infection experiment. (XLSX)

S1 Text Nucleotide sequences of phylogenetic tree isolates. (DOCX)
