# Supplementary material for: Isolation, identification and phenotypic and molecular characterization of pathogenic Vibrio vulnificus isolated from Litopenaeus vannamei
Source: PLoS One. 2017 Oct 18;12(10):e0186135. doi: 10.1371/journal.pone.0186135 (PMC5646798; doi:10.1371/journal.pone.0186135)
Supplement: S1 Table — (PDF) [file pone.0186135.s001.pdf]

Animal Care and Use Certification of Wuxi Fisheries College, Nanjing Agricultural University

Apply date: May 5, 2016

Applicant Name	Liguo Liang	Applicant organization	Wuxi Fisheries College of Nanjing Agricultural University
Contact Name	Liguo Liang	Phone	+8651085556566

Name of experiment: Isolation, Identification and Phenotypic and Molecular Characterization of Pathogenic *Vibrio vulnificus* Isolated from *Litopenaeus vannamei*

Experiment source: "The China Agriculture Research System special projectthe National Staple Freshwater Fish Industrial Technology System (CARS-46-10)" and "Aquaculture project in Jiangsu Province (D2015-13)".

Experiment department: The department of fish disease and nutrition

	Name	Position	Phone	Signature
Participants	Liguo Liang	Assistant professor	+8651085556566	Liguo Liang
	Jun Xie	Professor	+8651085559939	Jun Xie
	Pao Xu	Professor	+8651085557959	Pao Xu
	Bingwen Xi	Associate professor	+8651085556566	Bingwen Xi
	Tao Teng	Doctoral candidate	+8651085556566	Tao Teng
	Kai Chen	Teacher	+8651085556566	kai chen

Situation of using laboratory animal	Source	Incubated at Freshwater Fisheries Research Center, Chinese Academy of Fishery Sciences		
	Species	*Litopenaeus vannamei*	Grade	Common level
	Specifications	12±1 g	Quantity	460
	Entering date: May 5, 2016		Finishing date: June 1, 2016	

Experiment overview (including: experiment objective, methods, parameter, execute methods)

Experiment objective: the present study was aimed to select the pathogenic bacteria and determine the strength of its pathogenicity, according to the survival of *L. vannamei*.

Methods: ten groups of 10 *L. vannamei* each were infected with 10 suspected pathogenic bacteria isolated from dying *L. vannamei*, to select which is the pathogenic bacteria. After that, healthy *L. vannamei* were challenged by bath immersion with pure pathogenic bacteria cultures to test bacterial pathogenicity. The concentration of bacteria was 1×10^8, 5×10^7, 1×10^7, 5×10^6 and 1×10^6 CFU·mL^{-1}, respectively. Each concentration contained three groups of 20 shrimp each and the control group was established with sterile TSB liquid medium using the same dose. After infecting, *L. vannamei* were monitored for mortality within 7 days.

Parameters to determine: different survival of *L. vannamei* in 7 days.

Execute methods: *L. vannamei* were infected with *V. vulnificus* by bath immersion.

Project review

1. Whether the experiment should be carried out with animal, can it conduct with inanimate methods such as computer simulation and cell culture, or use lower animals instead of higher animals?
   (this experiment was specially designed with live animal, and cannot replace with other methods)
2. Whether the species and grade was suitable for this experiment? Was the number of animals be reduced by improving experiment design?
   (this is an optimal experiment design and used the least number of *L. vannamei*)
3. Whether it was kindness to animals by improving experiment methods, adjusting observation indexes, and modifying the treatments of animals?
   (the bath immersion was preceded by standard euthanasia)
4. Whether animal welfare measures implemented?
   (the animal welfare measures were implemented under the Animal Care and Use Committee of Nanjing Agricultural University (Nanjing, China))

Recommendation of Animal Care and Use Community

The animal welfare and care in this experiment were implemented under the instruction and received inspection of the Animal Care and Use Committee of Nanjing Agricultural University (Nanjing, China) (NJYY20160528-1). All animal procedures were performed according to the Guideline for the Care and Use of Laboratory Animals in China.

Signature: 梁利国 Liguo Liang

Date: May 5, 2016
