# Supplementary material for: Isolation, identification and phenotypic and molecular characterization of pathogenic Vibrio vulnificus isolated from Litopenaeus vannamei
Source: PLoS One. 2017 Oct 18;12(10):e0186135. doi: 10.1371/journal.pone.0186135 (PMC5646798; doi:10.1371/journal.pone.0186135)
Supplement: S1 Text — (DOCX) [file pone.0186135.s003.docx]

**S1 Nucleotide sequences of phylogenetic tree isolates**

These sequences of 19 strains are used to construct phylogenetic trees, and the nucleotide sequences are shown below:

**>GYX2014-7**

**GCCATGCTATCGCCTACACATGCAGTCGTGCAGCAGTCAACCCGAAACTTGTTTCTCGGGTGGCGAGCGGCGGACGGGTGAGTAATGCCTGGGAAATTGCCCTGATGTGGGGGATAACCATTGGAAACGATGGCTAATACCGCATGATGCCTACGGGCCAAAGAGGGGGACCTTCGGGCCTCTCGCGTCAGGATATGCCCAGGTGGGATTAGCTAGTTGGTGAGGTAAGGGCTCACCAAGGCGACGATCCCTAGCTGGTCTGAGAGGATGATCAGCCACACTGGAACTGAGACACGGTCCAGACTCCTACGGGAGGCAGCAGTGGGGAATATTGCACAATGGGCGCAAGCCTGATGCAGCCATGCCGCGTGTGTGAAGAAGGCCTTCGGGTTGTAAAGCACTTTCAGTTGTGAGGAAGGTGGTGTCGTTAATAGCGGCATCATTTGACGTTAGCAACAGAAGAAGCACCGGCTAACTCCGTGCCAGCAGCCGCGGTAATACGGAGGGTGCGAGCGTTAATCGGAATTACTGGGCGTAAAGCGCATGCAGGTGGTTTGTTAAGTCAGATGTGAAAGCCCGGGGCTCAACCTCGGAACTGCATTTGAAACTGGCAGACTAGAGTACTGTAGAGGGGGGTAGAATTTCAGGTGTAGCGGTGAAATGCGTAGAGATCTGAAGGAATACCGGTGGCGAAGGCGGCCCCCTGGACAGATACTGACACTCAGATGCGAAAGCGTGGGGAGCAAACAGGATTAGATACCCTGGTAGTCCACGCTGTAAACGATGTCTACTTGGAGGTTGTGGCCTTGAGCCGTGGCTTTCGGAGCTAACGCGTTAAGTAGACCGCCTGGGGAGTACGGTCGCAAGATTAAAACTCAAATGAATTGACGGGGGCCCGCACAAGCGGTGGAGCATGTGGTTTAATTCGATGCAACGCGAAGAACCTTACCTACTCTTGACATCCAGAGAATCTAGCGGAGACGCTGGAGTGCCTTCGGGAACTCTGAGACAGGTGCTGCATGGCTGTCGTCAGCTCGTGTTGTGAAATGTTGGGTTAAGTCCCGCAACGAGCGCAACCCTTATCCTTGTTTGCCAGCGAGTAATGTCGGGAACTCCAGGGAGACTGCCGGTGATAAACCGGAGGAAGGTGGGGACGACGTCAAGTCATCATGGCCCTTACGAGTAGGGCTACACACGTGCTACAATGGCGCATACAGAGGGCGGCCAACTTGCGAAAGTGAGCGAATCCCAAAAAGTGCGTCGTAGTCCGGATTGGAGTCTGCAACTCGACTCCATGAAGTCGGAATCGCTAGTAATCGTGGATCAGAATGCCACGGTGAATACGTTCCCGGGCCTTGTACACACCGCCCGTCACACCATGGGAGTGGGCTGCAAAAGAAGTGGGATGTAGTTAGCTCCTGGCTAGAGATCTCTAGAAAAACAGAGAA**

>JQ307112 *Vibrio vulnificus*

TTTGAACGCTGGCGGCAGGCCTAACACATGCAAGTCGAGCGGCAGCACAGAGAAACTTGTTTCTCGGGTGGCGAGCGGCGGACGGGTGAGTAATGCCTGGGAAATTGCCCTGATGTGGGGGATAACCATTGGAAACGATGGCTAATACCGCATGATGCCTACGGGCCAAAGAGGGGGACCTTCGGGCCTCTCGCGTCAGGATATGCCCAGGTGGGATTAGCTAGTTGGTGAGGTAAGGGCTCACCAAGGCGACGATCCCTAGCTGGTCTGAGAGGATGATCAGCCACACTGGAACTGAGACACGGTCCAGACTCCTACGGGAGGCAGCAGTGGGGAATATTGCACAATGGGCGCAAGCCTGATGCAGCCATGCCGCGTGTGTGAAGAAGGCCTTCGGGTTGTAAAGCACTTTCAGTTGTGAGGAAGGTGGTGTCGTTAATAGCGGCATCATTTGACGTTAGCAACAGAAGAAGCACCGGCTAACTCCGTGCCAGCAGCCGCGGTAATACGGAGGGTGCGAGCGTTAATCGGAATTACTGGGCGTAAAGCGCATGCAGGTGGTTTGTTAAGTCAGATGTGAAAGCCCGGGGCTCAACCTCGGAACTGCATTTGAAACTGGCAGACTAGAGTACTGTAGAGGGGGGTAGAATTTCAGGTGTAGCGGTGAAATGCGTAGAGATCTGAAGGAATACCGGTGGCGAAGGCGGCCCCCTGGACAGATACTGACACTCAGATGCGAAAGCGTGGGGAGCAAACAGGATTAGATACCCTGGTAGTCCACGCTGTAAACGATGTCTACTTGGAGGTTGTGGCCTTGAGCCGTGGCTTTCGGAGCTAACGCGTTAAGTAGACCGCCTGGGGAGTACGGTCGCAAGATTAAAACTCAAATGAATTGACGGGGGCCCGCACAAGCGGTGGAGCATGTGGTTTAATTCGATGCAACGCGAAGAACCTTACCTACTCTTGACATCCAGAGAATCCAGCGGAGACGCCGGAGTGCCTTCGGGAACTCTGAGACAGGTGCTGCATGGCTGTCGTCAGCTCGTGTTGTGAAATGTTGGGTTAAGTCCCGCAACGAGCGCAACCCTTATCCTTGTTTGCCAGCGAGTAATGTCGGGAACTCCAGGGAGACTGCCGGTGATAAACCGGAGGAAGGTGGGGACGACGTCAAGTCATCATGGCCCTTACGAGTAGGGCTACACACGTGCTACAATGGCGCATACAGAGGGCCGCCAACTTGCGAAAGTGAGCGAATCCCAAAAAGTGCGTCGTAGTCCGGATTGGAGTCTGCAACTCGACTCCATGAAGTCGGAATCGCTAGTAATCGTGGATCAGAATGCCACGGTGAATACGTTCCCGGGCCTTGTACACACCGCCCGTCACACCATGGGAGTGGGCTGCAAAAGAAGTGGGTAGTTTAACCTTCGGGAGGACGCTCACCACTTTGTGGTTCATGACTGGGGTGAAAAAA

>HQ380019 *Vibrio vulnificus*

CGGGCGTGCGGGCAGGTCTACACATGCAGTCGAGCGGCAGCACAGAGAAACTTGTTTCTCGGGTGGCGAGCGGCGGACGGGTGAGTAATGCCTGGGAAATTGCCCTGATGTGGGGGATAACCATTGGAAACGATGGCTAATACCGCATGATGCCTACGGGCCAAAGAGGGGGACCTTCGGGCCTCTCGCGTCAGGATATGCCCAGGTGGGATTAGCTAGTTGGTGAGGTAAGGGCTCACCAAGGCGACGATCCCTAGCTGGTCTGAGAGGATGATCAGCCACACTGGAACTGAGACACGGTCCAGACTCCTACGGGAGGCAGCAGTGGGGAATATTGCACAATGGGCGCAAGCCTGATGCAGCCATGCCGCGTGTGTGAAGAAGGCCTTCGGGTTGTAAAGCACTTTCAGTTGTGAGGAAGGTGGTGTCGTTAATAGCGGCATCATTTGACGTTAGCAACAGAAGAAGCACCGGCTAACTCCGTGCCAGCAGCCGCGGTAATACGGAGGGTGCGAGCGTTAATCGGAATTACTGGGCGTAAAGCGCATGCAGGTGGTTTGTTAAGTCAGATGTGAAAGCCCGGGGCTCAACCTCGGAACTGCATTTGAAACTGGCAGACTAGAGTACTGTAGAGGGGGGTAGAATTTCAGGTGTAGCGGTGAAATGCGTAGAGATCTGAAGGAATACCGGTGGCGAAGGCGGCCCCCTGGACAGATACTGACACTCAGATGCGAAAGCGTGGGGAGCAAACAGGATTAGATACCCTGGTAGTCCACGCTGTAAACGATGTCTACTTGGAGGTTGTGGCCTTGAGCCGTGGCTTTCGGAGCTAACGCGTTAAGTAGACCGCCTGGGGAGTACGGTCGCAAGATTAAAACTCAAATGAATTGACGGGGGCCCGCACAAGCGGTGGAGCATGTGGTTTAATTCGATGCAACGCGAAGAACCTTACCTACTCTTGACATCCAGAGAAGCCAGCGGAGACGCAGGTGTGCCTTCGGGAACTCTGAGACAGGTGCTGCATGGCTGTCGTCAGCTCGTGTTGTGAAATGTTGGGTTAAGTCCCGCAACGAGCGCAACCCTTATCCTTGTTTGCCAGCGAGTAATGTCGGGAACTCCAGGGAGACTGCCGGTGATAAACCGGAGGAAGGTGGGGACGACGTCAAGTCATCATGGCCCTTACGAGTAGGGCTACACACGTGCTACAATGGCGCATACAGAGGGCGGCCAACTTGCGAAAGTGAGCGAATCCCAAAAAGTGCGTCGTAGTCCGGATTGGAGTCTGCAACTCGACTCCATGAAGTCGGAATCGCTAGTAATCGTGGATCAGAATGCCACGGTGAATACGTTCCCGGGCCTTGTACACACCGCCCGTCACACCATGGGAGTGGGCTGCAAAAGAAGTGGGTAGTTTAACCTTCGGGAGGACGCTCACCACTTAAGTCGGGCG

>KC748469 *Vibrio vulnificus*

TGGGGAGCTACCATGCAGTCGAGCGGCAGCACAGAGAAACTTGTTTCTCGGGTGGCGAGCGGCGGACGGGTGAGTAATGCCTGGGAAATTGCCCTGATGTGGGGGATAACCATTGGAAACGATGGCTAATACCGCATGATGCCTACGGGCCAAAGAGGGGGACCTTCGGGCCTCTCGCGTCAGGATATGCCCAGGTGGGATTAGCTAGTTGGTGAGGTAAGGGCTCACCAAGGCGACGATCCCTAGCTGGTCTGAGAGGATGATCAGCCACACTGGAACTGAGACACGGTCCAGACTCCTACGGGAGGCAGCAGTGGGGAATATTGCACAATGGGCGCAAGCCTGATGCAGCCATGCCGCGTGTGTGAAGAAGGCCTTCGGGTTGTAAAGCACTTTCAGTTGTGAGGAAGGTGGTGTCGTTAATAGCGGCATCATTTGACGTTAGCAACAGAAGAAGCACCGGCTAACTCCGTGCCAGCAGCCGCGGTAATACGGAGGGTGCGAGCGTTAATCGGAATTACTGGGCGTAAAGCGCATGCAGGTGGTTTGTTAAGTCAGATGTGAAAGCCCGGGGCTCAACCTCGGAACTGCATTTGAAACTGGCAGACTAGAGTACTGTAGAGGGGGGTAGAATTTCAGGTGTAGCGGTGAAATGCGTAGAGATCTGAAGGAATACCGGTGGCGAAGGCGGCCCCCTGGACAGATACTGACACTCAGATGCGAAAGCGTGGGGAGCAAACAGGATTAGATACCCTGGTAGTCCACGCTGTAAACGATGTCTACTTGGAGGTTGTGGCCTTGAGCCGTGGCTTTCGGAGCTAACGCGTTAAGTAGACCGCCTGGGGAGTACGGTCGCAAGATTAAAACTCAAATGAATTGACGGGGGCCCGCACAAGCGGTGGAGCATGTGGTTTAATTCGATGCAACGCGAAGAACCTTACCTACTCTTGACATCCAGAGAAGCCAGCGGAGACGCAGGTGTGCCTTCGGGAACTCTGAGACAGGTGCTGCATGGCTGTCGTCAGCTCGTGTTGTGAAATGTTGGGTTAAGTCCCGCAACGAGCGCAACCCTTATCCTTGTTTGCCAGCGAGTAATGTCGGGAACTCCAGGGAGACTGCCGGTGATAAACCGGAGGAAGGTGGGGACGACGTCAAGTCATCATGGCCCTTACGAGTAGGGCTACACACGTGCTACAATGGCGCATACAGAGGGCGGCCAACTTGCGAAAGTGAGCGAATCCCAAAAAGTGCGTCGTAGTCCGGATTGGAGTCTGCAACTCGACTCCATGAAGTCGGAATCGCTAGTAATCGTGGATCAGAATGCCACGGTGAATACGTTCCCGGGCCTTGTACACACCGCCCGTCACACCATGGGAGTGGGCTGCAAAAGAAGTGGGTAGTTTAACCTTCGGGAGGACGCTCACCACTTTTGTACGC

>JQ433550 *Vibrio vulnificus*

AGAGTTTGATCCAGGCTCAGATTGAACGCTGGCGGCAGGCCTAACACATGCAAGTCGAGCGGCAGCACAGAGAAACTTGTTTCTCGGGTGGCGAGCGGCGGACGGGTGAGTAATGCCTGGGAAATTGCCCTGATGTGGGGGATAACCATTGGAAACGATGGCTAATACCGCATGATGCCTACGGGCCAAAGAGGGGGACCTTCGGGCCTCTCGCGTTAGGATATGCCCAGGTGGGATTAGCTAGTTGGTGAGGTAAGGGCTCACCAAGGCGACGATCCCTAGCTGGTCTGAGAGGATGATCAGCCACACTGGAACTGAGACACGGTCCAGACTCCTACGGGAGGCAGCAGTGGGGAATATTGCACAATGGGCGCAAGCCTGATGCAGCCATGCCGCGTGTGTGAAGAAGGCCTTCGGGTTGTAAAGCACTTTCAGTTGTGAGGAAGGTGGTGTCGTTAATAGAGGCATCATTTGACGTTAGCAACAGAAGAAGCACCGGCTAACACCGTGCCAGCAGCCGCGGTAATACGGAGGGTGCGAGCGTTAATCGGAATTACTGGGCGTAAAGCGCATGCAGGTGGTTTGTTAAGTCAGATGTGAAAGCCCGGGGCTCAACCTCGGAACTGCATTTGAAACTGGCAGACTAGAGTACTGTAGAGGGGGGTAGAATTTCAGGTGTAGCGGTGAAAATGCGTAGAGATCTGAAGGAATACCGGTGGCGAAGGCGGCCCCCTGGACAGATACTGACACTCAGATGCGAAAGCGTGGGGAGCAAACAGGATTAGATACCCTGGTAGTCCACGCTGTAAACGATGTCTACTTGGAGGTTGTGGCCTTGAGCCGTGGCTTTCGGAGCTAACGCGTTAAGTAGACCGCCTGGGGAGTACGGTCGCAAGATTAAAACTCAAATGAATTGACGGGGGCCCGCACAAGCGGTGGAGCATGTGGTTTAATTCGATGCAACGCGAAGAACCTTACCTACTCTTGACATCCAGAGAACCTAGCGGAGACGCTGGAGTGCCTTCGGGAACTCTGAGACAGGTGCTGCATGGCTGTCGTCAGCTCGTGTTGTGAAATGTTGGGTTAAGTCCCGCAACGAGCGCAACCCTTATCCTTGTTTGCCAGCGAGTAATGTCGGGAACTCCAGGGAGACTGCCGGTGATAAACCGGAGGAAGGTGGGGACGACGTCAAGTCATCATGGCCCTTACGAGTAGGGCTACACACGTGCTACAATGGCGCATACAGAGGGCGGCCAACTTGCGAAAGTGAGCGAATCCCAAAAAGTGCGTCGTAGTCCGGATTGGAGTCTGCAACTCGACTCCATGAAGTCGGAATCGCTAGTAATCGTGGATCAGAATGCCACGGTGAATACGTTCCCGGGCCTTGTACACACCGCCCGTCACACCATGGGAGTGGGCTGCAAAAGAAGTGGGTAGTTTAACCTTCGGGAGGACGCTCACCACTTTGTGGTTCATGACTGGGGTGAAGTCCTAACAAGGTAA

>EU931029 *Vibrio vulnificus*

GCCTGGGGGGGCTGCTAACACATGCAAGTCGAGCGGCAGCACAGAGAAACTTGTTTCTCGGGTGGCGAGCGGAGGACGGGTGAGTAATGCCTGGGAAATTGCCCTGATGTGGGGGATAACCATTGGAAACGATGGCTAATACCGCATGATGCCTACGGGCCAAAGAGGGGGACCTTCGGGCCTCTCGCGTCAGGATATGCCCAGGTGGGATTAGCTAGTTGGTGAGGTAAGGGCTCACCAAGGCGACGATCCCTAGCTGGTCTGAGAGGATGATCAGCCACACTGGAACTGAGACACGGTCCAGACTCCTACGGGAGGCAGCAGTGGGGAATATTGCACAATGGGCGCAAGCCTGATGCAGCCATGCCGCGTGTGTGAAGAAGGCCTTCGGGTTGTAAAGCACTTTCAGTTGTGAGGAAGGTGGTGTCGTTAATAGCGGCATCATTTGACGTTAGCAACAGAAGAAGCACCGGCTAACTCCGTGCCAGCAGCCGCGGTAATACGGAGGGTGCGAGCGTTAATCGGAATTACTGGGCGTAAAGCGCATGCAGGTGGTTTGTTAAGTCAGATGTGAAAGCCCGGGGCTCAACCTCGGAACTGCATTTGAAACTGGCAGACTAGAGTACTGTAGAGGGGGGTAGAATTTCAGGTGTAGCGGTGAAATGCGTAGAGATCTGAAGGAATACCGGTGGCGAAGGCGGCCCCCTGGACAGATACTGACACTCAGATGCGAAAGCGTGGGGAGCAAACAGGATTAGATACCCTGGTAGTCCACGCTGTAAACGATGTCTACTTGGAGGTTGTGGCCTTGAGCCGTGGCTTTCGGAGCTAACGCGTTAAGTAGACCGCCTGGGGAGTACGGTCGCAAGATTAAAACTCAAATGAATTGACGGGGGCCCGCACAAGCGGTGGAGCATGTGGTTTAATTCGATGCAACGCGAAGAACCTTACCTACTCTTGACATCCAGAGAAGCCAGCGGAGACGCAGGTGTGCCTTCGGGAACTCTGAGACAGGTGCTGCATGGCTGTCGTCAGCTCGTGTTGTGAAATGTTGGGTTAAGTCCCGCAACGAGCGCAACCCTTATCCTTGTTTGCCAGCGAGTAATGTCGGGAACTCCAGGGAGACTGCCGGTGATAAACCGGAGGAAGGTGGGGACGACGTCAAGTCATCATGGCCCTTACGAGTAGGGCTACACACGTGCTACAATGGCGCATACAGAGGGCGGCCAACTTGCGAAAGTGAGCGAATCCCAAAAAGTGCGTCGTAGTCCGGATTGGAGTCTGCAACTCGACTCCATGAAGTCGGAATCGCTAGTAATCGTGGATCAGAATGCCACGGTGAATACGTTCCCGGGCCTTGTACACACCGCCCGTCACACCATGGGAGTGGGCTGCAAAAGAAGTGGGTAGTTTAACCTTCGGGAGGACGCTCACGCACGGGGGGGGCA

>KC884613 *Vibrio vulnificus*

GAGCGGCAGCACAGAGAAACTTGTTTCTCGGGTGGCGAGCGGCGGACGGGTGAGTAATGCCTGGGAAATTGCCCTGATGTGGGGGATAACCATTGGAAACGATGGCTAATACCGCATGATGCCTACGGGCCAAAGAGGGGGACCTTCGGGCCTCTCGCGTCAGGATATGCCCAGGTGGGATTAGCTAGTTGGTGAGGTAAGGGCTCACCAAGGCGACGATCCCTAGCTGGTCTGAGAGGATGATCAGCCACACTGGAACTGAGACACGGTCCAGACTCCTACGGGAGGCAGCAGTGGGGAATATTGCACAATGGGCGCAAGCCTGATGCAGCCATGCCGCGTGTGTGAAGAAGGCCTTCGGGTTGTAAAGCACTTTCAGTTGTGAGGAAGGTGGTGTCGTTAATAGCGGCATCATTTGACGTTAGCAACAGAAGAAGCACCGGCTAACTCCGTGCCAGCAGCCGCGGTAATACGGAGGGTGCGAGCGTTAATCGGAATTACTGGGCGTAAAGCGCATGCAGGTGGTTTGTTAAGTCAGATGTGAAAGCCCGGGGCTCAACCTCGGAACTGCATTTGAAACTGGCAGACTAGAGTACTGTAGAGGGGGGTAGAATTTCAGGTGTAGCGGTGAAATGCGTAGAGATCTGAAGGAATACCGGTGGCGAAGGCGGCCCCCTGGACAGATACTGACACTCAGATGCGAAAGCGTGGGGAGCAAACAGGATTAGATACCCTGGTAGTCCACGCTGTAAACGATGTCTACTTGGAGGTTGTGGCCTTGAGCCGTGGCTTTCGGAGCTAACGCGTTAAGTAGACCGCCTGGGGAGTACGGTCGCAAGATTAAAACTCAAATGAATTGACGGGGGCCCGCACAAGCGGTGGAGCATGTGGTTTAATTCGATGCAACGCGAAGAACCTTACCTACTCTTGACATCCAGAGAAGCCAGCGGAGACGCAGGTGTGCCTTCGGGAACTCTGAGACAGGTGCTGCATGGCTGTCGTCAGCTCGTGTTGTGAAATGTTGGGTTAAGTCCCGCAACGAGCGCAACCCTTATCCTTGTTTGCCAGCGAGTAATGTCGGGAACTCCAGGGAGACTGCCGGTGATAAACCGGAGGAAGGTGGGGACGACGTCAAGTCATCATGGCCCTTACGAGTAGGGCTACACACGTGCTACAATGGCGCATACAGAGGGCGGCCAACTTGCGAAAGTGAGCGAATCCCAAAAAGTGCGTCGTAGTCCGGATTGGAGTCTGCAACTCGACTCCATGAAGTCGGAATCGCTAGTAATCGTGGATCAGAATGCCACGGTGAATACGTTCCCGGGCCTTGTACACACCGCCCGTCACACCATGGGAGTGGGCTGCAAAAGAAGTGGGTAGTTTAACCTTCGGGAGGACGCTCACCACTT

>KP058475 *Vibrio vulnificus*

CACGCATAAGCGAAGCTACCATGCAGTCGAGCGGCAGCACAGAGAAACTTGTTTCTCGGGTGGCGAGCGGCGGACGGGTGAGTAATGCCTGGGAAATTGCCCTGATGTGGGGGATAACCATTGGAAACGATGGCTAATACCGCATGATGCCTACGGGCCAAAGAGGGGGACCTTCGGGCCTCTCGCGTCAGGATATGCCCAGGTGGGATTAGCTAGTTGGTGAGGTAAGGGCTCACCAAGGCGACGATCCCTAGCTGGTCTGAGAGGATGATCAGCCACACTGGAACTGAGACACGGTCCAGACTCCTACGGGAGGCAGCAGTGGGGAATATTGCACAATGGGCGCAAGCCTGATGCAGCCATGCCGCGTGTGTGAAGAAGGCCTTCGGGTTGTAAAGCACTTTCAGTTGTGAGGAAGGTGGTGTCGTTAATAGCGGCATCATTTGACGTTAGCAACAGAAGAAGCACCGGCTAACTCCGTGCCAGCAGCCGCGGTAATACGGAGGGTGCGAGCGTTAATCGGAATTACTGGGCGTAAAGCGCATGCAGGTGGTTTGTTAAGTCAGATGTGAAAGCCCGGGGCTCAACCTCGGAACTGCATTTGAAACTGGCAGACTAGAGTACTGTAGAGGGGGGTAGAATTTCAGGTGTAGCGGTGAAATGCGTAGAGATTTGAAGGAATACCGGTGGCGAAGGCGGCCCCCTGGACAGATACTGACACTCAGATGCGAAAGCGTGGGGAGCAAAACAGGATTAGATACCCTGGTAGTCCACGCTGTAAACGATGTCTACTTGGAGGTTGTGGCCTTGAGCCGTGGCTTTCGGAGCTAACGCGTTAAGTAGACCGCCTGGGGAGTACGGTCGCAAGATTAAAACTCAAATGAATTGACGGGGGCCCGCACAAGCGGTGGAGCATGTGGTTTAATTCGATGCAACGCGAAGAACCTTACCTACTCTTGACATCCAGAGAAGCCAGCGGAGACGCAGGTGTGCCTTCGGGAACTCTGAGACAGGTGCTGCATGGCTGTCGTCAGCTCGTGTTGTGAAATGTTGGGTTAAGTCCCGCAACGAGCGCAACCCTTATCCTTGTTTGCCAGCGAGTAATGTCGGGAACTCCAGGGAGACTGCCGGTGATAAACCGGAGGAAGGTGGGGACGACGTCAAGTCATCATGGCCCTTACGAGTAGGGCTACACACGTGCTACAATGGCGCATACAGAGGGCGGCCAACTTGCGAAAGTGAGCGAATCCCAAAAAGTGCGTCGTAGTCCGGATTGGAGTCTGCAACTCGACTCCATGAAGTCGGAATCGCTAGTAATCGTGGATCAGAATGCCACGGTGAATACGTTCCCGGGCCTTGTACACACCGCCCGTCACACCATGGGAGTGGGCTGCAAAAGAAGTGGGTAGTTTAACCTTCGGGAGGACGCTCACCACTTTGGGCGTGCCTT

>AY676130 *Vibrio vulnificus*

AGGCCTTAACACTCGCAAGTCGAGCGGCAGCACAGAGAAACTTGTTTCTCAGGTGGCGAGCGGCGGACGGGTGAGTAATGCCTGGGAAATTGCCCTGATGTGGGGGATAACCATTGGAAACGATGGCTAATACCGCATGATGCCTACGGGCCAAAGAGGGGGACCTTCGGGCCTCTCGCGTCAGGATATGCCCAGGTGGGATTAGCTAGTTGGTGAGGTAAGGGCTCACCAAGGCGACGATCCCTAGCTGGTCTGAGAGGATGATCAGCCACACTGGAACTGAGACACGGTCCAGACTCCTACGGGAGGCAGCAGTGGGGAATATTGCACAATGGGCGCAAGCCTGATGCAGCCATGCCGCGTGTGTGAAGAAGGCCTTCGGGTTGTAAAGCACTTTCAGTTGTGAGGAAGGTGGTGTCGTTAATAGCGGCATCATTTGACGTTAGCAACAGAAGAAGCACCGGCTAACTCCGTGCCAGCAGCCGCGGTAATACGGAGGGTGCGAGCGTTAATCGGAATTACTGGGCGTAAAGCGCATGCAGGTGGTTTGTTAAGTCAGATGTGAAAGCCCGGGGCTCAACCTCGGAACTGCATTTGAAACTGGCAGACTAGAGTACTGTAGAGGGGGGTAGAATTTCAGGTGTAGCGGTGAAATGCGTAGAGATCTGAAGGAATACCGGTGGCGAAGGCGGCCCCTGGACAGATACTGACACTCAGATGCGAAAGCGTGGGGAGCAAACAGGATTAGATACCCTGGTAGTCCACGCTGTAAACGATGTCTACTTGGAGGTTGTGGCCTTGACCGTGCTTTCGGAGCTAACGCGTTAAGTAGACCGCCTGGGGAGTACGGTCGCAAGATTAAAACTCAAATGAATTGACGGGGGCCCGCACAAGCGGTGGAGCATGTGGTTTAATTCGATGCAACGCGAAGAACCTTACCTACTCTTGACATCCAGAGAAGCCAGCGGAGACGCAGGTGTGCCTTCGGGAACTCTGAGACAGGTGCTGCATGGCTGTCGTCAGCTCGTGTTGTGAAATGTTGGGTTAAGTCCCGCAACGAGCGCAACCCTTATCCTTGTTTGCCAGCGAGTAATGTCGGGAACTCCAGGGAGACTGCCGGTGATAAACCGGAGGAAGGTGGGGACGACGTCAAGTCATCATGGCCCTTACGAGTAGGGCTACACACGTGCTACAATGGCGCATACAGAGGGCGGCCAACTTGCGAAAGTGAGCGAATCCCAAAAAGTGCGTCGTAGTCCGGATTGGAGTCTGCAACTCGACTCCATGAAGTCGGAATCGCTAGTAATCGTGGATCAGAATGCCACGGTGAATACGTTCCCGGGCCTTGTACACACCGCCCGTCACACCATGGGAGTGGGCTGCAAAAGAAGTGGGTAGTTTAACCTTCGGGTAGGCGCCTCGACCCACTTTGTGGTTCAATGACTGGGTCAAGGGTATCA

>NR_113792 *Vibrio vulnificus*

ATTGAACGCTGGCGGCAGGCCTAACACATGCAAGTCGAGCGGCAGCACAGAGAAACTTGTTTCTCGGGTGGCGAGCGGCGGACGGGTGAGTAATGCCTGGGAAATTGCCCTGATGTGGGGGATAACCATTGGAAACGATGGCTAATACCGCATGATAGCTTCGGCTCAAAGAGGGGGACCTTCGGGCCTCTCGCGTCAGGATATGCCCAGGTGGGATTAGCTAGTTGGTGAGGTAAGGGCTCACCAAGGCGACGATCCCTAGCTGGTCTGAGAGGATGATCAGCCACACTGGAACTGAGACACGGTCCAGACTCCTACGGGAGGCAGCAGTGGGGAATATTGCACAATGGGCGCAAGCCTGATGCAGCCATGCCGCGTGTGTGAAGAAGGCCTTCGGGTTGTAAAGCACTTTCAGTCGTGAGGAAGGTGGTAGTGTTAATAGCACTATCATTTGACGTTAGCGACAGAAGAAGCACCGGCTAACTCCGTGCCAGCAGCCGCGGTAATACGGAGGGTGCGAGCGTTAATCGGAATTACTGGGCGTAAAGCGCATGCAGGTGGTTTGTTAAGTCAGATGTGAAAGCCCGGGGCTCAACCTCGGAACTGCATTTGAAACTGGCAGACTAGAGTACTGTAGAGGGGGGTAGAATTTCAGGTGTAGCGGTGAAATGCGTAGAGATCTGAAGGAATACCGGTGGCGAAGGCGGCCCCCTGGACAGATACTGACACTCAGATGCGAAAGCGTGGGGAGCAAACAGGATTAGATACCCTGGTAGTCCACGCTGTAAACGATGTCTACTTGGAGGTTGTGGCCTTGAGCCGTGGCTTTCGGAGCTAACGCGTTAAGTAGACCGCCTGGGGAGTACGGTCGCAAGATTAAAACTCAAATGAATTGACGGGGGCCCGCACAAGCGGTGGAGCATGTGGTTTAATTCGATGCAACGCGAAGAACCTTACCTACTCTTGACATCCAGAGAATCTAGCGGAGACGCTGGAGTGCCTTCGGGAACTCTGAGACAGGTGCTGCATGGCTGTCGTCAGCTCGTGTTGTGAAATGTTGGGTTAAGTCCCGCAACGAGCGCAACCCTTATCCTTGTTTGCCAGCGAGTAATGTCGGGAACTCCAGGGAGACTGCCGGTGATAAACCGGAGGAAGGTGGGGACGACGTCAAGTCATCATGGCCCTTACGAGTAGGGCTACACACGTGCTACAATGGCGCATACAGAGGGCGGCCAACTTGCGAAAGTGAGCGAATCCCAAAAAGTGCGTCGTAGTCCGGATTGGAGTCTGCAACTCGACTCCATGAAGTCGGAATCGCTAGTAATCGTGGATCAGAATGCCACGGTGAATACGTTCCCGGGCCTTGTACACACCGCCCGTCACACCATGGGAGTGGGCTGCAAAAGAAGTGGGTAGTTTAACCTTCGGGAGGACGCTCACCACTTTGTGGTTCATGACTGGGGTGAAG

>KC884604 *Vibrio vulnificus*

AACACATGCAAGTCGAGCGGCAGCACAGAGAAACTTGTTTCTCGGGTGGCGAGCGGCGGACGGGTGAGTAATGCCTGGGAAATTGCCCTGATGTGGGGGATAACCATTGGAAACGATGGCTAATACCGCATGATAGCTTCGGCTCAAAGAGGGGGACCTTCGGGCCTCTCGCGTCAGGATATGCCCAGGTGGGATTAGCTAGTTGGTGAGGTAAGGGCTCACCAAGGCGACGATCCCTAGCTGGTCTGAGAGGATGATCAGCCACACTGGAACTGAGACACGGTCCAGACTCCTACGGGAGGCAGCAGTGGGGAATATTGCACAATGGGCGCAAGCCTGATGCAGCCATGCCGCGTGTGTGAAGAAGGCCTTCGGGTTGTAAAGCACTTTCAGTCGTGAGGAAGGTGGTAGTGTTAATAGCACTATCATTTGACGTTAGCGACAGAAGAAGCACCGGCTAACTCCGTGCCAGCAGCCGCGGTAATACGGAGGGTGCGAGCGTTAATCGGAATTACTGGGCGTAAAGCGCATGCAGGTGGTTTGTTAAGTCAGATGTGAAAGCCCGGGGCTCAACCTCGGAACTGCATTTGAAACTGGCAGACTAGAGTACTGTAGAGGGGGGTAGAATTTCAGGTGTAGCGGTGAAATGCGTAGAGATCTGAAGGAATACCGGTGGCGAAGGCGGCCCCCTGGACAGATACTGACACTCAGATGCGAAAGCGTGGGGAGCAAACAGGATTAGATACCCTGGTAGTCCACGCTGTAAACGATGTCTACTTGGAGGTTGTGGCCTTGAGCCGTGGCTTTCGGAGCTAACGCGTTAAGTAGACCGCCTGGGGAGTACGGTCGCAAGATTAAAACTCAAATGAATTGACGGGGGCCCGCACAAGCGGTGGAGCATGTGGTTTAATTCGATGCAACGCGAAGAACCTTACCTACTCTTGACATCCAGAGAATCTAGCGGAGACGCTGGAGTGCCTTCGGGAACTCTGAGACAGGTGCTGCATGGCTGTCGTCAGCTCGTGTTGTGAAATGTTGGGTTAAGTCCCGCAACGAGCGCAACCCTTATCCTTGTTTGCCAGCGAGTAATGTCGGGAACTCCAGGGAGACTGCCGGTGATAAACCGGAGGAAGGTGGGGACGACGTCAAGTCATCATGGCCCTTACGAGTAGGGCTACACACGTGCTACAATGGCGCATACAGAGGGCGGCCAACTTGCGAAAGTGAGCGAATCCCAAAAAGTGCGTCGTAGTCCGGATTGGAGTCTGCAACTCGACTCCATGAAGTCGGAATCGCTAGTAATCGTGGATCAGAATGCCACGGTGAATACGTTCCCGGGCCTTGTACACACCGCCCGTCACACCATGGGAGTGGGCTGCAAAAGAAGTGGGTAGTTTAACCTTCGGGAGGACGCTCACCACTT

>NR_117906 *Vibrio vulnificus*

CAGGCCTAACACATGCAAGTCGAGCGGCAGCACAGAGAAACTTGTTTCTCGGGTGGCGAGCGGCGGACGGGTGAGTAATGCCTGGGAAATTGCCCTGATGTGGGGGATAACCATTGGAAACGATGGCTAATACCGCATGATGCCTACGGGCCAAAGAGGGGGACCTTCGGGCCTCTCGCGTCAGGATATGCCCAGGTGGGATTAGCTAGTTGGTGAGGTAAGGGCTCACCAAGGCGACGATCCCTAGCTGGTCTGAGAGGATGATCAGCCACACTGGAACTGAGACACGGTCCAGACTCCTACGGGAGGCAGCAGTGGGGAATATTGCACAATGGGCGCAAGCCTGATGCAGCCATGCCGCGTGTGTGAAGAAGGCCTTCGGGTTGTAAAGCACTTTCAGTTGTGAGGAAGGTGGTGTCGTTAATAGCGGCATCATTTGACGTTAGCAACAGAAGAAGCACCGGCTAACTCCGTGCCAGCAGCCGCGGTAATACGGAGGGTGCGAGCGTTAATCGGAATTACTGGGCGTAAAGCGCATGCAGGTGGTTTGTTAAGTCAGATGTGAAAGCCCGGGGCTCAACCTCGGAACTGCATTTGAAACTGGCAGACTAGAGTACTGTAGAGGGGGGTAGAATTTCAGGTGTAGCGGTGAAATGCGTAGAGATCTGAAGGAATACCGGTGGCGAAGGCGGCCCCCTGGACAGATACTGACACTCAGATGCGAAAGCGTGGGGAGCAAACAGGATTAGATACCCTGGTAGTCCACGCTGTAAACGATGTCTACTTGGAGGTTGTGGCCTTGAGCCGTGGCTTTCGGAGCTAACGCGTTAAGTAGACCGCCTGGGGAGTACGGTCGCAAGATTAAAACTCAAATGAATTGACGGGGGCCCGCACAAGCGGTGGAGCATGTGGTTTAATTCGATGCAACGCGAAGAACCTTACCTACTCTTGACATCCAGAGAATCTAGCGGAGACGCTGGAGTGCCTTCGGGAACTCTGAGACAGGTGCTGCATGGCTGTCGTCAGCTCGTGTTGTGAAATGTTGGGTTAAGTCCCGCAACGAGCGCAACCCTTATCCTTGTTTGCCAGCGAGTAATGTCGGGAACTCCAGGGAGACTGCCGGTGATAAACCGGAGGAAGGTGGGGACGACGTCAAGTCATCATGGCCCTTACGAGTAGGGCTACACACGTGCTACAATGGCGCATACAGAGGGCAGCCAACTTGCGAAAGTGAGCGAATCCCAAAAAGTGCGTCGTAGTCCGGATTGGAGTCTGCAACTCGACTCCATGAAGTCGGAATCGCTAGTAATCGTGGATCAGAATGCCACGGTGAATACGTTCCCGGGCCTTGTACACACCGCCC

>KF886645 *Vibrio vulnificus*

AGTCGAGCGGCAGCACAGAGAAACTTGTTTCTCGGGTGGCGAGCGGCGGACGGGTGAGTAATGCCTGGGAAATTGCCCTGATGTGGGGGATAACCATTGGAAACGATGGCTAATACCGCATGATGCCTACGGGCCAAAGAGGGGGACCTTCGGGCCTCTCGCGTCAGGATATGCCCAGGTGGGATTAGCTAGTTGGTGAGGTAAGGGCTCACCAAGGCGACGATCCCTAGCTGGTCTGAGAGGATGATCAGCCACACTGGAACTGAGACACGGTCCAGACTCCTACGGGAGGCAGCAGTGGGGAATATTGCACAATGGGCGCAAGCCTGATGCAGCCATGCCGCGTGTGTGAAGAAGGCCTTCGGGTTGTAAAGCACTTTCAGTTGTGAGGAAGGTGGTGTCGTTAATAGCGGCATCATTTGACGTTAGCAACAGAAGAAGCACCGGCTAACTCCGTGCCAGCAGCCGCGGTAATACGGAGGGTGCGAGCGTTAATCGGAATTACTGGGCGTAAAGCGCATGCAGGTGGTTTGTTAAGTCAGATGTGAAAGCCCGGGGCTCAACCTCGGAACTGCATTTGAAACTGGCAGACTAGAGTACTGTAGAGGGGGGTAGAATTTCAGGTGTAGCGGTGAAATGCGTAGAGATCTGAAGGAATACCGGTGGCGAAGGCGGCCCCCTGGACAGATACTGACACTCAGATGCGAAAGCGTGGGGAGCAAACAGGATTAGATACCCTGGTAGTCCACGCTGTAAACGATGTCTACTTGGAGGTTGTGGCCCTGAGCCGTGGCTTTTGGAGCTAACGCGTTAAGTAGACCGCCTGGGGAGTACGGTCGCAAGATTAAACTCAAATGAATTGACGGGGGCCCGCACAAGCGGGGAGCATGTGGTTTAATTCGATGCAACGCGAAGAACCTTACCTACTCTTGACATCCAGAGAAGCCAGCGGAGACGCAGGTGTGCCTTCGGGAACTTTGAGACAGGTGCTGCATGGCTGTCGTCAGCTCGTGTTGTGAAATGTTGGGTTAAGTCCCGCAACGAGCGCAACCCTTATCCTTGTTTGCCAGCGAGTAATGTCGGGAACTCCAGGGAGACTGCCGGTGATAAACCGGAGGAAGGTGGGGACGACGTCAAGTCATCATGGCCCTTACGAGTAGGGCTACACACGTGCTACAATGGCGCATACAGAGGGCGGCCAACTTGCGAAAGTGAGCGAATCCCAAAAAGTGCGTCGTAGTCCGGATTGGAGTTTGCAACTCGACTCCCTGAAGTCGGAATCGCTAGTAATCGTGGATCAGAATGCCCCGGTGAATACGTTCCCGGGCCTTGTACACACCGCCCGTCACACCATGGGAGTGGGCTGCAAAAGAAGTGGGTAGTTAACCTCGGGAGGACGCTCACCCACT

>NR_118570 *Vibrio vulnificus*

CTTGTTTCTCGGGTGGCGAGCGGCGGACGGGTGAGTAATGCCTGGGAAATTGCCCTGATGTGGGGGATAACCATTGGAAACGATGGCTAATACCGCATGATAGCTTCGGCTCAAAGAGGGGGACCTTCGGGCCTCTCGCGTCAGGATATGCCCAGGTGGGATTAGCTAGTTGGTGAGGTAAGGGCTCACCAAGGCGACGATCCCTAGCTGGTCTGAGAGGATGATCAGCCACACTGGAACTGAGACACGGTCCAGACTCCTACGGGAGGCAGCAGTGGGGAATATTGCACAATGGGCGCAAGCCTGATGCAGCCATGCCGCGTGTGTGAAGAAGGCCTTCGGGTTGTAAAGCACTTTCAGTCGTGAGGAAGGTGGTAGTGTTAATAGCACTATCATTTGACGTTAGCGACAGAAGAAGCACCGGCTAACTCCGTGCCAGCAGCCGCGGTAATACGGAGGGTGCGAGCGTTAATCGGAATTACTGGGCGTAAAGCGCATGCAGGTGGTTTGTTAAGTCAGATGTGAAAGCCCGGGGCTCAACCTCGGAACTGCATTTGAAACTGGCAGACTAGAGTACTGTAGAGGGGGGTAGAATTTCAGGTGTAGCGGTGAAATGCGTAGAGATCTGAAGGAATACCGGTGGCGAAGGCGGCCCCCTGGACAGATACTGACACTCAGATGCGAAAGCGTGGGGAGCAAACAGGATTAGATACCCTGGTAGTCCACGCTGTAAACGATGTCTACTTGGAGGTTGTGGCCTTGAGCCGTGGCTTTCGGAGCTAACGCGTTAAGTAGACCGCCTGGGGAGTACGGTCGCAAGATTAAAACTCAAATGAATTGACGGGGGCCCGCACAAGCGGTGGAGCATGTGGTTTAATTCGATGCAACGCGAAGAACCTTACCTACTCTTGACATCCAGAGAATCTAGCGGAGACGCTGGAGTGCCTTCGGGAACTCTGAGACAGGTGCTGCATGGCTGTCGTCAGCTCGTGTTGTGAAATGTTGGGTTAAGTCCCGCAACGAGCGCAACCCTTATCCTTGTTTGCCAGCGAGTAATGTCGGGAACTCCAGGGAGACTGCCGGTGATAAACCGGAGGAAGGTGGGGACGACGTCAAGTCATCATGGCCCTTACGAGTAGGGCTACACACGTGCTACAATGGCGCATACAGAGGGCGGCCAACTTGCGAAAGTGAGCGAATCCCAAAAAGTGCGTCGTAGTCCGGATTGGAGTCTGCAACTCGACTCCATGAAGTCGGAATCGCTAGTAATCGTGGATCAGAATGCCACGGTGAATACGTTCCCGGGCCTTGTACACACCGCCCGTCACACCATGGGAGTGGGCTGCAAAAGAAGTGGGTAG

>JQ253967 *Vibrio vulnificus*

CAAGTCGAGCGGCAGCACAGAGAAACTTGTTTCTCGGGTGGCGAGCGGCGGACGGGTGAGTAATGCCTGGGAAATTGCCCTGATGTGGGGGATAACCATTGGAAACGATGGCTAATACCGCATGATAGCTTCGGCTCAAAGAGGGGGACCTTCGGGCCTCTCGCGTCAGGATATGCCCAGGTGGGATTAGCTAGTTGGTGAGGTAAGGGCTCACCAAGGCGACGATCCCTAGCTGGTCTGAGAGGATGATCAGCCACACTGGAACTGAGACACGGTCCAGACTCCTACGGGAGGCAGCAGTGGGGAATATTGCACAATGGGCGCAAGCCTGATGCAGCCATGCCGCGTGTGTGAAGAAGGCCTTCGGGTTGTAAAGCACTTTTCAGTCGTGAGGAAGGTGGTAGTGTTAATAGCACTATCATTTGACGTTAGCGACAGAAGAAGCACCGGGCTAACTCCGTGCCAGCAGCCGCGGTAATACGGAGGGTGCGAGCGTTAATCGGAATTACTGGGCGTAAAGCGCATGCAGGTGGTTTGTTAAGTCAGATGTGAAAGCCCGGGGCTCAACCTCGGAACTGCATTTGAAACTGGCAGACTAGAGTACTGTAGAGGGGGGTAGAATTTCAGGTGTAGCGGTGAAATGCGTAGAGATCTGAAGGAATACCGGTGGCGAAGGCGGCCCCCTGGACAGATACTGACACTCAGATGCGAAAGCGTGGGGAGCAAACAGGATTAGATACCCTGGTAGTCCACGCTGTAAACGATGTCTACTTGGAGGTTGTGGCCTTGAGCCGTGGCTTTCGGAGCTAACGCGTTAAGTAGACCGCCTGGGGAGTACGGTCGCAAGATTAAAACTCAAATGAATTGACGGGGGCCCGCACAAGCGGTGGAGCATGTGGTTTAATTCGATGCAACGCGAAGAACCTTACCTACTCTTGACATCCAGAGAATCTAGCGGAGACGCTGGAGTGCCTTCGGGAACTCTGAGACAGGTGCTGCATGGCTGTCGTCAGCTCGTGTTGTGAAATGTTGGGTTAAGTCCCGCAACGAGCGCAACCCTTATCCTTGTTTGCCAGCGAGTAATGTCGGGAACTCCAGGGAGACTGCCGGTGATAAACCGGAGGAAGGTGGGGACGACGTCAAGTCATCATGGCCCTTACGAGTAGGGCTACACACGTGCTACAATGGCGCATACAGAGGGCGGCCAACTTGCGAAAGTGAGCGAATCCCAAAAAGTGCGTCGTAGTCCGGATTGGAGTCTGCAACTCGACTCCATGAAGTCGGAATCGCTAGTAATCGTGGATCAGAATGCCACGGTGAATACGTTCCCGGGCCTTGTACACACCGCCCGTCACACCATGGGAGTGGGCTGCAAAAGAAGTGGGTAGTTTAAC

>KT074371 *Vibrio vulnificus*

CACATGCAAGTCGAGCGGCAGCACAGAGAAACTTGTTTCTCGGGTGGCGAGCGGCGGACGGGTGAGTAATGCCTGGGAAATTGCCCTGATGTGGGGGATAACCATTGGAAACGATGGCTAATACCGCATGATAGCTTCGGCTCAAAGAGGGGGACCTTCGGGCCTCTCGCGTCAGGATATGCCCAGGTGGGATTAGCTAGTTGGTGAGGTAAGGGCTCACCAAGGCGACGATCCCTAGCTGGTCTGAGAGGATGATCAGCCACACTGGAACTGAGACACGGTCCAGACTCCTACGGGAGGCAGCAGTGGGGAATATTGCACAATGGGCGCAAGCCTGATGCAGCCATGCCGCGTGTGTGAAGAAGGCCTTCGGGTTGTAAAGCACTTTCAGTCGTGAGGAAGGTGGTAGTGTTAATAGCACTATCATTTGACGTTAGCGACAGAAGAAGCACCGGCTAACTCCGTGCCAGCAGCCGCGGTAATACGGAGGGTGCGAGCGTTAATCGGAATTACTGGGCGTAAAGCGCATGCAGGTGGTTTGTTAAGTCAGATGTGAAAGCCCGGGGCTCAACCTCGGAACTGCATTTGAAACTGGCAGACTAGAGTACTGTAGAGGGGGGTAGAATTTCAGGTGTAGCGGTGAAATGCGTAGAGATCTGAAGGAATACCGGTGGCGAAGGCGGCCCCCTGGACAGATACTGACACTCAGATGCGAAAGCGTGGGGAGCAAACAGGATTAGATACCCTGGTAGTCCACGCTGTAAACGATGTCTACTTGGAGGTTGTGGCCTTGAGCCGTGGCTTTCGGAGCTAACGCGTTAAGTAGACCGCCTGGGGAGTACGGTCGCAAGATTAAAACTCAAATGAATTGACGGGGGCCCGCACAAGCGGTGGAGCATGTGGTTTAATTCGATGCAACGCGAAGAACCTTACCTACTCTTGACATCCAGAGAATCTAGCGGAGACGCTGGAGTGCCTTCGGGAACTCTGAGACAGGTGCTGCATGGCTGTCGTCAGCTCGTGTTGTGAAATGTTGGGTTAAGTCCCGCAACGAGCGCAACCCTTATCCTTGTTTGCCAGCGAGTAATGTCGGGAACTCCAGGGAGACTGCCGGTGATAAACCGGAGGAAGGTGGGGACGACGTCAAGTCATCATGGCCCTTACGAGTAGGGCTACACACGTGCTACAATGGCGCATACAGAGGGCGGCCAACTTGCGAAAGTGAGCGAATCCCAAAAAGTGCGTCGTAGTCCGGATTGGAGTCTGCAACTCGACTCCATGAAGTCGGAATCGCTAGTAATCGTGGATCAGAATGCCACGGTGAATACGTTCCCGGGCCTTGTACACACCGCCCGTCACACCATGGGAGTGGGCTGC

>KC291508 *Vibrio vulnificus*

GAGAAACTTGTTTCATCGGGTGGCGAGCGGCGGACGGGTGAGTAATGCCTGGGAAATTGCCCTGATGTGGGGGATAACCATTGGAAACGATGGCTAATACCGCATGATGCCTACGGGCCAAAGAGGGGGACCTTCGGGCCTCTCGCGTCAGGATATGCCCAGGTGGGATTAGCTAGTTGGTGAGGTAAGGGCTCACCAAGGCGACGATCCCTAGCTGGTCTGAGAGGATGATCAGCCACACTGGAACTGAGACACGGTCCAGACTCCTACGGGAGGCAGCAGTGGGGAATATTGCACAATGGGCGCAAGCCTGATGCAGCCATGCCGCGTGTGTGAAGAAGGCCTTCGGGTTGTAAAGCACTTTCAGTTGTGAGGAAGGTGGTGTCGTTAATAGCGGCATCATTTGACGTTAGCAACAGAAGAAGCACCGGCTAACTCCGTGCCAGCAGCCGCGGTAATACGGAGGGTGCGAGCGTTAATCGGAATTACTGGGCGTAAAGCGCATGCAGGTGGTTTGTTAAGTCAGATGTGAAAGCCCGGGGCTCAACCTCGGAACTGCATTTGAAACTGGCAGACTAGAGTACTGTAGAGGGGGGTAGAATTTCAGGTGTAGCGGTGAAATGCGTAGAGATCTGAAGGAATACCGGTGGCGAAGGCGGCCCCCTGGACAGATACTGACACTCAGATGCGAAAGCGTGGGGAGCAAACAGGATTAGATACCCTGGTAGTCCACGCTGTAAACGATGTCTACTTGGAGGTTGTGGCCTTGAGCCGTGGCTTTCGGAGCTAACGCGTTAAGTAGACCGCCTGGGGAGTACGGTCGCAAGATTAAAACTCAAATGAATTGACGGGGGCCCGCACAAGCGGTGGAGCATGTGGTTTAATTCGATGCAACGGCGGAAGAATCCTTACCTACTCTTTGACATCCAGAGAATGCCTAGCGGAGAACGCAGGTAGTGCCTTCGGGAACTCTTGAGAACAGGTGCTGCATGGCTTGTCGGTCAGCTCGTGTTTGTGAAATGTTGGGTTAAGTCCCGCAACGAGCGCAACCCTTATCCTTGTTTGCCAGCGAGTAATGTCGGGAACTCCAGGGAGACTGCCGGTGATAAACCGGAGGAAGGTGGGGACGACGTCAAGTCATCATGGCCCTTACGAGTAGGGCTACACACGTGCTACAATGGCGCATACAGAGGGCGGCCAACTTGCGAAAGTGAGCGAATCCCAAAAAGTGCGTCGTAGTCCGGATTGGAGTCTGCAACTCGACTCCATGAAGTCGGAATCGCTAGTAATCGTGGATCAGAATGCCAGGTGAATACGTTCCCGGGCCTTGTACACACCGCCGTCACACCATGGGAGTGGGCTGCAAAAGAAGTGGGTAGTTTAACCTTCGGGAGGACGCTCACCACTTTGTGGTTCATGA

>JQ307113 *Vibrio vulnificus*

TTTGAACGCTGGCGGCAGGCCTAACACATGCAAGTCGAGCGGCAGCACAGAGAAACTTGTTTCTCGGGGGGCGAGCGGCGGACGGGGGAGTAATGCCTGGGAAATTGCCCTGATGTGGGGGATAACCATTGGAAACGATGGCTAATACCGCATGATGCCTACGGGCCAAAGAGGGGGACCTTCGGGCCTCTCGCGTCAGGATATGCCCAGGTGGGATTAGCTAGTTGGGGAGGTAAGGGCTCACCAAGGCGACGATCCCTAGCTGGTCTGAGAGGATGATCAGCCACACTGGAACTGAGACACGGTCCAGACTCCTACGGGAGGCAGCAGTGGGGAATATTGCACAATGGGCGCAAGCCTGATGCAGCCATGCCGCGTGTGTGAAGAAGGCCTTCGGGTTGTAAAGCACTTTCAGTTGTGAGGAAGGGGGTGTCGTTAATAGCGGCATCATTTGACGTTAGCAACAGAAGAAGCACCGGCTAACTCCGTGCCAGCAGCCGCGGTAATACGGAGGGGGCGAGCGTTAATCGGAATTACTGGGCGTAAAGCGCATGCAGGGGGTTTGTTAAGTCAGATGTGAAAGCCCGGGGCTCAACCTCGGAACTGCATTTGAAACTGGCAGACTAGAGTACTGTAGAGGGGGGTAGAATTTCAGGGGTAGCGGGGAAATGCGTAGAGATCTGAAGGAATACCGGGGGCGAAGGCGGCCCCCTGGACAGATACTGACACTCAGATGCGAAAGCGTGGGGAGCAAACAGGATTAGATACCCTGGTAGTCCACGCTGTAAACGATGTCTACTTGGAGGTTGTGGCCTTGAGCCGTGGCTTTCGGAGCTAACGCGTTAAGTAGACCGCCTGGGGAGTACGGTCGCAAGATTAAAACTCAAATGAATTGACGGGGGCCCGCACAAGCGGGGGAGCATGTGGTTTAATTCGATGCAACGCGAAGAACCTTACCTACTCTTGACATCCAGAGAATCCAGCGGAGACGCCGGAGTGCCTTCGGGAACTCTGAGACAGGGGCTGCATGGCTGTCGTCAGCTCGTGTTGTGAAATGTTGGGTTAAGTCCCGCAACGAGCGCAACCCTTATCCTTGTTTGCCAGCGAGTAATGTCGGGAACTCCAGGGAGACTGCCGGGGATAAACCGGAGGAAGGGGGGGACGACGTCAAGTCATCATGGCCCTTACGAGTAGGGCTACACACGTGCTACAATGGCGCATACAGAGGGCCGCCAACTTGCGAAAGTGAGCGAATCCCAAAAAGTGCGTCGTAGTCCGGATTGGAGTCTGCAACTCGACTCCATGAAGTCGGAATCGCTAGTAATCGTGGATCAGAATGCCACGGGGAATACGTTCCCGGGCCTTGTACACACCGCCCGTCACACCATGGGAGTGGGCTGCAAAAGAAGTGGGTAGTTTAACCTTCGGGAGGACGCTCACCACTTTGTGGTTCATGACTGGGGGGAAAAAA

>KF386612 *Vibrio cholerae*

GGGGGGCGGGCCTAACACATGCAAGTCGAGCGGCAGCACAGAGGAACTTGTTCCTTGGGTGGCGAGCGGCGGACGGGTGAGTAATGCCTGGGAAATTGCCCGGTAGAGGGGGATAACCATTGGAAACGATGGCTAATACCGCATAACCTCGCAAGAGCAAAGCAGGGGACCTTCGGGCCTTGCGCTACCGGATATGCCCAGGTGGGATTAGCTAGTTGGTGAGGTAAGGGCTCACCAAGGCGACGATCCCTAGCTGGTCTGAGAGGATGATCAGCCACACTGGAACTGAGACACGGTCCAGACTCCTACGGGAGGCAGCAGTGGGGAATATTGCACAATGGGCGCAAGCCTGATGCAGCCATGCCGCGTGTATGAAGAAGGCCTTCGGGTTGTAAAGTACTTTCAGTAGGGAGGAAGGTGGTTAAGTTAATACCTTAATCATTTGACGTTACCTACAGAAGAAGCACCGGCTAACTCCGTGCCAGCAGCCGCGGTAATACGGAGGGTGCAAGCGTTAATCGGAATTACTGGGCGTAAAGCGCATGCAGGTGGTTTGTTAAGTCAGATGTGAAAGCCCTGGGCTCAACCTAGGAATCGCATTTGAAACTGACAAGCTAGAGTACTGTAGAGGGGGGTAGAATTTCAGGTGTAGCGGTGAAATGCGTAGAGATCTGAAGGAATACCGGTGGCGAAGGCGGCCCCCTGGACAGATACTGACACTCAGATGCGAAAGCGTGGGGAGCAAACAGGATTAGATACCCTGGTAGTCCACGCCGTAAACGATGTCTACTTGGAGGTTGTGCCCTAGAGGCGTGGCTTTCGGAGCTAACGCGTTAAGTAGACCGCCTGGGGAGTACGGTCGCAAGATTAAAACTCAAATGAATTGACGGGGGCCCGCACAAGCGGTGGAGCATGTGGTTTAATTCGATGCAACGCGAAGAACCTTACCTACTCTTGACATCCAGAGAATCTAGCGGAGACGCTGGAGTGCCTTCGGGAGCTCTGAGACAGGTGCTGCATGGCTGTCGTCAGCTCGTGTTGTGAAATGTTGGGTTAAGTCCCGCAACGAGCGCAACCCTTATCCTTGTTTGCCAGCACGTAATGGTGGGAACTCCAGGGAGACTGCCGGTGATAAACCGGAGGAAGGTGGGGACGACGTCAAGTCATCATGGCCCTTACGAGTAGGGCTACACACGTGCTACAATGGCGTATACAGAGGGCAGCGATACCGCGAGGTGGAGCGAATCTCACAAAGTACGTCGTAGTCCGGATTGGAGTCTGCAACTCGACTCCATGAAGTCGGAATCGCTAGTAATCGCAAATCAGAATGTTGCGGTGAATACGTTCCCGGGCCTTGTACACACCGCCCGTCACACCATGGGAGTGGGCTGCAAAAGAAGCAGGTAGTTTAACCTTCGGGAGGACGCTGCCACTTTTGGCC
